# Thermoreversible Gels Based on Chitosan Copolymers as “Intelligent” Drug Delivery System with Prolonged Action for Intramuscular Injection

**DOI:** 10.3390/pharmaceutics15051478

**Published:** 2023-05-12

**Authors:** Igor D. Zlotnikov, Stanislav M. Malashkeevich, Natalia G. Belogurova, Elena V. Kudryashova

**Affiliations:** Faculty of Chemistry, Lomonosov Moscow State University, Leninskie Gory, 1/3, 119991 Moscow, Russia

**Keywords:** polycationic polymer, thermoreversible gel, gene delivery, DNA polyplex, flow cytometry, FTIR

## Abstract

Thermosensitive gels based on copolymers (PEG–chitosan, chitosan–polyethylenimine, chitosan–arginine and glycol–chitosan–spermine) are presented as promising polycations for the formation of DNA polyplexes and the potential for the development of drugs with prolonged release (up to 30 days). Being in liquid form at room temperature, such compounds can be injected into muscle tissue with rapid gel formation at human body temperature. An intramuscular depot is formed with a therapeutic agent that provides a gradual release of the drug, such as an antibacterial or cytostatic. The physico-chemical parameters of the formation of polyplexes between polycationic polymers of various compositions and molecular architecture and DNA were studied via FTIR, UV-vis and fluorescence spectroscopy using the dyes rhodamine 6G (R6G) and acridine orange (AO). The competitive displacement of AO from AO-DNA complexes showed that, with a ratio of N/P = 1, most of the DNA is bound to a polycation. During the formation of polyplexes, the DNA charge is neutralized by a polycation, which is reflected in electrophoretic immobility. The cationic polymers described in this work at a concentration of 1–4% are capable of forming gels, and the thermoreversible property is most characteristic of pegylated chitosan. BSA, as a model anionic molecule, is released by half in 5 days from the Chit5-PEG5 gel; full release is achieved in 18–20 days. At the same time, in 5 days, the gel is destroyed up to 30%, and in 20 days, by 90% (release of chitosan particles). For the first time, flow cytometry was used to study DNA polyplexes, which showed the existence of fluorescent particles in a much larger number in combination with free DNA. Thus, functional stimulus-sensitive polymers are potentially applicable for the creation of prolonged therapeutic formulations for gene delivery systems, which were obtained. The revealed regularities appear to be a platform for the design of polyplexes with controllable stability, in particular, fulfilling the requirements imposed for gene delivery vehicles.

## 1. Introduction

Thermoreversible gels based on biocompatible and biodegradable polymers, such as chitosan and polyethylene glycol (PEG), are promising for biomedical use. Thermoreversible gels can be used to deliver drugs and nucleic acids with the function of prolonged action during intramuscular release. Thermoreversible polymers are liquid at room temperature and form a gel at physiological temperatures [1,2,3,4]. These polymers are promising in the field of creating combined drug delivery systems, including delivery of genetic material into cells.

Gene therapy is promising in the treatment of genetic diseases, including lower limb ischemia, cardiovascular diseases, cystic fibrosis and arthritis [5,6,7,8]. Nucleic acids (RNA and DNA) must be delivered to the intracellular medium, and in the case of plasmid DNA, (pDNA) to the cell nucleus. The nucleic acid delivery system is necessary to prevent the destruction of DNA by nucleases and increase the efficiency of transfection. Viral vectors transfect DNA most effectively, but they are expensive and there are problems with production and safety. Promising transfecting agents are polycations, for example, polyethylenimine (PEI) [5,6,9,10,11,12,13,14], but having a cytotoxic effect. Therefore, in order to use polycations in gene delivery, it is necessary to modify them with bio-friendly molecules: polyethylene glycol, chitosan and arginine. Cationic polymers can electrostatically interact with negatively charged nucleic acids to form polyplexes. Natural polymers (cationic collagen derivatives, dextrin, polysaccharides, poly (glycolic acid), poly (lactic acid), chitosan [5,6,11,15,16]), as gene delivery vehicles, in comparison with other cationic polymers, have a higher charge density, quickly releasing DNA after internalization, which leads to more efficient gene transfection. The use of unmodified chitosan is limited by its poor solubility under physiological conditions (pKa ≅ 6.4) and low transfection activity in vitro. Therefore, water-soluble modified chitosans are used in our work, and a modification with arginine (positive charge) and gluconic acid with the use of glycol–chitosan is proposed. Conjugates based on the gold standard of transfection of PEI and chitosan are even more effective, while reducing negative effects and increasing transfection [5,6,11].

Copolymers of polyethylene glycol (PEG), poly (lactic acid) and chitosan are biodegradable and promising nucleic acid delivery systems. PEG is a neutral, water-soluble, synthetic but non-toxic polymer, approved by the FDA in food products, cosmetics, personal care products and pharmaceutical preparations. PEG is widely used as a component in gene/drug delivery systems [2,8,12,16,17,18,19,20,21,22,23,24,25,26,27]. Chitosan–PEG conjugates are the most prominent representatives of thermoreversible gels that can be used to treat genetic diseases, for example, lower limb ischemia, when an intramuscular gel should release DNA for about 20–40 days [2].

Thus, this work is devoted to the development of a drug/gene delivery system based on the thermal sensitive copolymers in the aspects of prolonged release studied on the model polyanions: DNA or/and negatively charged protein (BSA) for medical use.

## 2. Materials and Methods

### 2.1. Reagents

Chitosan oligosaccharide lactate 5 kDa (Chit5), glycolchitosan 72 kDa (GlicChit72), spermine (sp), carbonyldiimidazole (CDI), polyethyleneimine 1.8 kDa (PEI1.8), 1-Ethyl-3-(3-dimethylaminopropyl) carbodiimide (EDC), N-hydroxysuccinimide (NHS) and 1M 2,4,6-trinitrobenzenesulfonic acid (TNBS) were obtained from Sigma Aldrich (St. Louis, MI, USA).

### 2.2. Synthesis of Polymers

The chemical conjugates of Chit5-Arg and Chit5-Arg-GlucA were synthesized via the coupling reaction of COOH group of acids with NH_2−_ group in the presence of 1-ethyl-3-(3-dimethylaminopropyl) carbodiimide (EDC) at 60 °C for 12 h. Arginine (Arg) 24 mg and gluconic acid (GlucA) 30 mg were dissolved separately in 0.5 mL of sodium acetate buffer (0.05 M, pH 5) and then activated with a mixture of 3-fold molar excess of EDC and 2-fold molar excess of N-Hydroxysuccinimide (NHS) in EtOH/buffer (pH 5, 50/50 *v*/*v*). Activated arginine was added to 70 mg Chit5 in 10 mL of sodium acetate buffer, and the mixture was incubated for 4 h at 60 °C. Then, dialysis treatment was performed against water for 6 h (MWCO 12–14 kDa), and the operation was repeated with the addition of activated GlucA.

Chit5-PEG5 conjugate was obtained through reaction within 4 h of Chit5 and activated PEG5 (N-succinimidyl ester of mono-methoxy poly(ethylene glycol)) 1 to 2 by weight in PBS (0.01 M, pH 7.4).

Chit5-PEI1.8 was synthesized using a crosslinking agent CDI. CDI was mixed with Chit5 (1/1 *w*/*w*), and mixture was dissolved in PBS followed by 1 h incubation at 40 °C. Then, PEI1.8 (0.2 from Chit5 mass) was added dropwise into reaction mixture followed by 4 h incubation at 60 °C.

GlicChit72-sp was synthesized similarly to Chit5-PEI1.8 with GlicChit72:sp = 6:1 mass ratio.

All samples were purified through dialysis against water (MWCO 12–14 kDa) for 6–12 h followed by freeze-drying at −70 °C (Edwards 5, BOC Edwards, Burgess Hill, UK). The grafting (modification) degree was estimated based on kinetic curves (A 420 from time) during titration of amino groups by 2,4,6-trinitrobenzenesulfonic acid (TNBS) using 1 M TNBS solution in 1 M sodium-borate buffer (pH 9.2). The calculation was carried out with respect to unmodified chitosan.

### 2.3. Isolation of pDNA of Small Plasmids for Analytical Experiments

Plasmid pBluescript II (2.9 kbase, Stratagene) with bacterial DNA insertion (4 kbase) was used to refine the technique. DNA of recombinant plasmids up to 10 kbase in size was isolated in accordance with the modified method of “Alkaline lysis” of Birnboim-Doly [28]. The resulting preparations are stored in the refrigerator at a temperature of −20 °C.

### 2.4. Synthesis of Polyplexes

Polyplexes were obtained by incubating a plasmid DNA solution with a polymer in various molar ratios at 37 °C. Fluorescent images of DNA-polymer gel particles were obtained using ZOE Fluorescent Cell Imager (Bio-Rad Laboratories, Hercules, CA, USA).

### 2.5. FTIR Spectroscopy

ATR-FTIR spectra of cell samples were recorded using a Bruker Tensor 27 spectrometer equipped with a liquid-nitrogen-cooled MCT (mercury cadmium telluride) detector. Samples were placed in a thermostatic cell BioATR-II with ZnSe ATR element (Bruker, Bremen, Germany). FTIR spectra were acquired from 850 to 4000 cm^−1^, with 1 cm^−1^ spectral resolution. For each spectrum, 50 scans were accumulated and averaged. Spectral data were processed using the Bruker software system Opus 8.2.28 (Bruker, Bremen, Germany).

### 2.6. Fluorescence and UV-Vis Spectroscopy

Registration of fluorescence spectra was carried out using a Varian Cary Eclipse spectrofluorometer (Agilent Technologies, Santa Clara, CA, USA). UV-visible spectra of AO, AO-DNA and AO-DNA + polymers were recorded on the AmerSham Biosciences UltraSpec 2100 pro device (Piscataway, NJ, USA), 0.5 mM HCl. Background spectrum was subtracted as a blank.

### 2.7. DNA Electrophoresis in Agarose Gel

We used an underwater (“submarine”) version of electrophoresis. The device BioRad Power Supply Model 250/2.5 served as a constant voltage source. In experiments, 0.8% agarose was used at E = 3 V/cm. A single buffer for electrophoresis contained: 40 mM tris-acetate; 20 mM sodium acetate; 2 mM EDTA; pH = 7.9; 0.5 mg/L of ethidium bromide. DNA samples were introduced into the wells of agarose gel as part of a single loading solution (5% glycerin, 10 mM EDTA, Ponceaus S “Reanal” dye).

### 2.8. Flow Cytometry

CytoFLEX S flow cytometer (Beckman Coulter, Brea, CA, USA) was used to study DNA-free and DNA polyplexes with AO. The CytoFLEX S flow cytometer was set up according to the manufacturer’s instructions. The sample tube was loaded onto the flow cytometer, and data were collected using the 488 nm laser for excitation.

The fluorescence emissions were collected using a 585/42 nm bandpass filter. Data were collected for 10,000 particles for each sample. The collected data were then analyzed using CytExpert software (Beckman Coulter, Inc., Miami, FL, USA). Gating was applied to exclude debris and doublets, and only single cells were analyzed. Mean fluorescence intensity (MFI) was measured.

### 2.9. Gel Formation in Muscle Tissue on Chicken Model

The study of gel properties in chicken muscle tissue was carried out using chilled meat on bones (chicken legs) with skin. The gel at a concentration of 0.25–1% was mixed with a 0.1% solution of tissue dye isosulfan blue (Lymphotropin) in PBS or 0.1 mg/mL with a solution of rhodamine 6G. The colored gel was injected at a temperature of 25 °C from a syringe to a depth of 0.5–1 cm under the skin of a chicken heated to 37 °C. Incubation at 37 °C was carried out for 2–24–48 h to study gel formation and spreading of the dye.

### 2.10. The Release of DNA and BSA from the Gel

Here, 20 mg of Chit5-PEG5 polymer was mixed with 5 mg of BSA and 0.2 mg of DNA, respectively, and the samples were dissolved in 1 mL of PBS (pH 7.4). The mixtures were intensively vortexed, followed by incubation at 37 °C for 30 min. Then, 1 mL of gels was placed inside the dialysis chamber (cut-off 1 MDA). Incubation at 37 °C was carried out for 30 days with the release of 10 mL into an external solution. Samples from the external solution were taken with subsequent registration of UV and IR spectra to determine the calibration dependence of the concentration of BSA, DNA and chitosan (gel structure unit). DNA was detected via A260. Chit5-PEG5 was detected via A235.

### 2.11. Statistical Analysis

Statistical analysis of experimental data was performed using the Student’s *t*-test Origin 2022 software (OriginLab Corporation, Northampton, MA, USA). Values are presented as the mean ± SD of three experiments (three replicates).

## 3. Results and Discussion

### 3.1. Synthesis of Cationic Copolymers Formed Thermoreversible Gels

**Figure 1:**
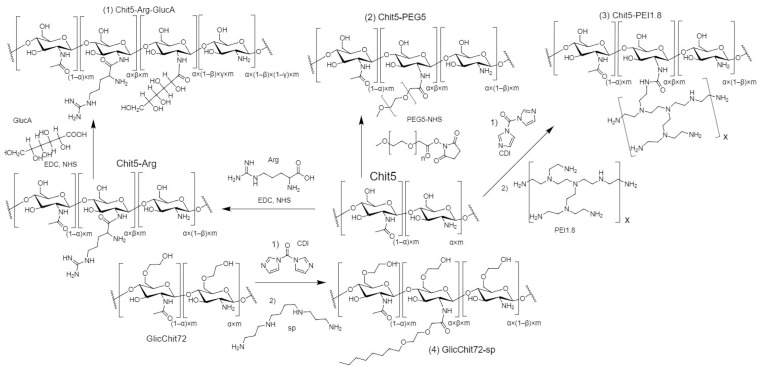
Scheme of synthesis of grafted chitosans.

To optimize the efficiency of binding with polyanions and thermoreversible properties, we selected grafted chitosans as polycations: (1) Chit5-Arg-GlucA (Arg for positive charge and GlucA for solubility), (2) Chit5-PEG5 (PEG—thermosensitive gel formation), (3) Chit5-PEI1.8 (PEI—“gold” reference polycation for transfection, but toxic and, therefore, modified with chitosan), (4) GlicChit72-sp (glycol modification of Chit72 for solubility and spermine (sp) for positive charge), as promising polymers that form polyplexes with DNA and effectively transfect into cells. The synthesis of polymers was carried out according to the scheme shown in Figure 1, using the reactions of the formation of an amide bond between the NH_2−_ group and the activated carboxyl group, the crosslinking of amino groups using carbonyldiimidazole.

### 3.2. Characterization of Cationic Copolymers

**Figure 2:**
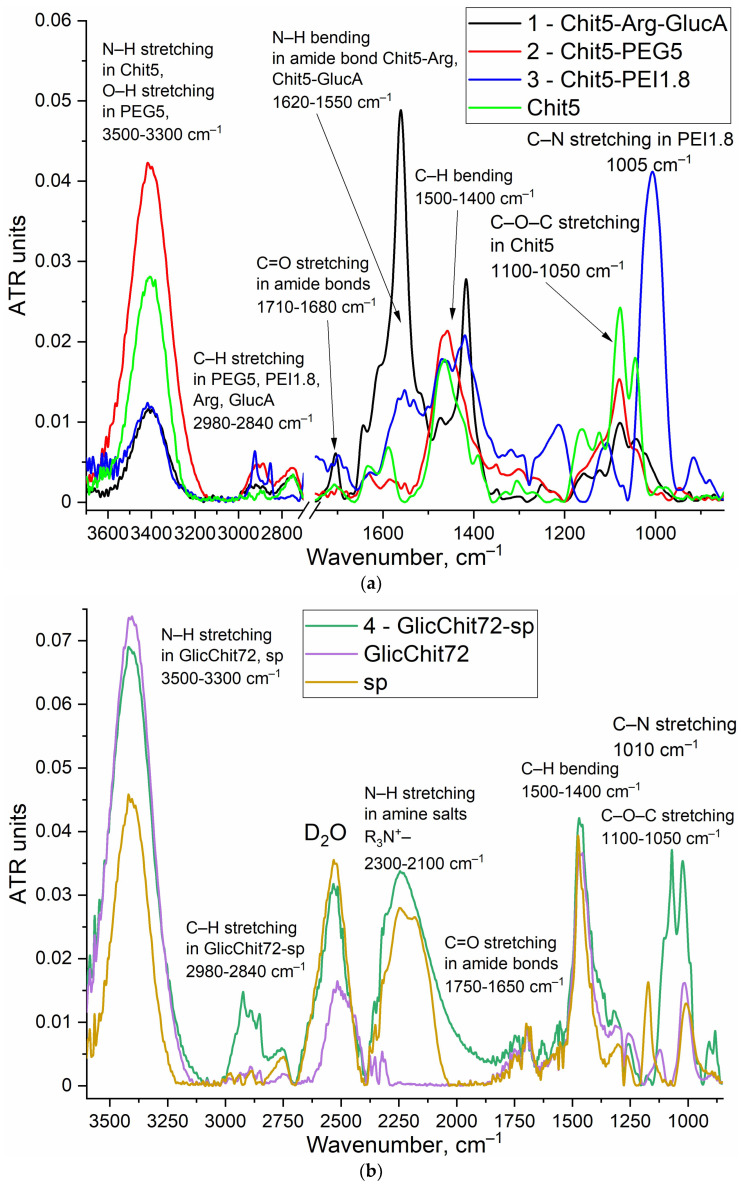

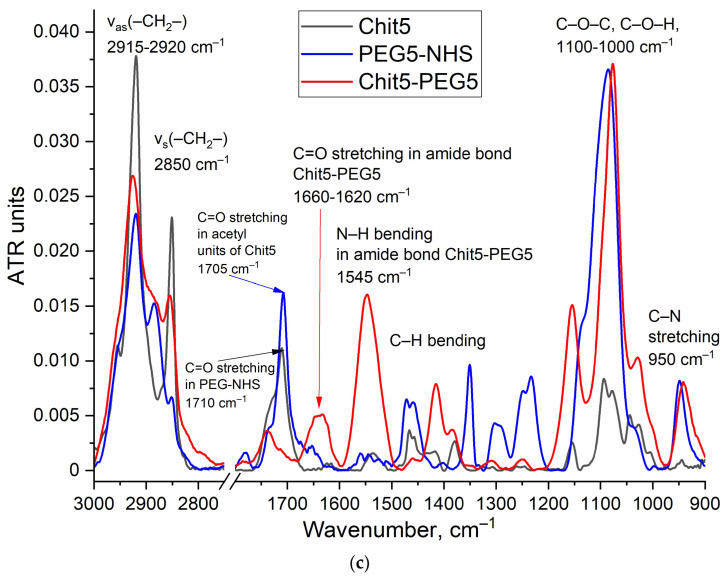
FTIR spectra of (**a**) Chit5 and three grafted Chit5 in D_2_O; (**b**) GlicChit72, sp and its conjugate GlicChit72-sp Chit5 in D_2_O; (**c**) Chit5, activated PEG5 (PEG5-NHS) and its conjugate Chit5-PEG5 in H_2_O. 22 °C.

The chemical structures of grafted chitosans were studied using FTIR and spectroscopy (Figure 2). Peaks of the initial components are represented in the spectra of conjugates 1–4 (Figure 2a,b): N–H and O–H stretching of Chit5 and PEG5 (3500–3300 cm^−1^), C–O–C stretching of Chit5 (1100–1050 cm^−1^), ν_s_ (C–H) and ν_as_ (C–H) at 2980–2840 cm^−1^. The conjugate’ 2 spectra are shown in Figure 2a (in D_2_O) and Figure 2c (H_2_O). The deuterated solvent makes it possible to study the oscillations of N–H and O–H in polymers, since they are not blocked by water (minor compared with water). However, the peaks of ν_s_ (C–H) and ν_as_ (C–H) are better manifested in water, the change in the shape and position of the maximum of which characterizes the formation of crosslinking between Chit5 and PEG5.

The conjugate’s formation was confirmed by a decrease in peak intensity of N–H stretching (Figure 2a) due to modification of chitosan amino groups; increases in peak intensity (Figure 2a–c) 1000 cm^−1^ (C–N), 1540–1620 cm^−1^ (amide –NH–) and 1660–1620 and 1710–1680 cm^−1^ (–C(=O)–) were due to the formation of amide bonds between chitosan and Arg/GlucA/PEG/PEI/sp.

Based on FTIR spectroscopy data (integral peak intensities), TNBS amino-group titration and initial component ratios, the average modification degrees and chemical composition of conjugates were determined (Table 1).

### 3.3. Phase Transition of Gel Formation Polymers

#### 3.3.1. FTIR Spectroscopy

One of the key aspects of this work is the study of the thermal sensitivity of gel-forming polymers. FTIR spectroscopy allows you to monitor the microenvironment of the main functional groups, which provides information about the molecular architecture of polymers and their folding (hydrophobic interactions/aggregation) when heated (thermoreversible properties).

**Figure 3:**
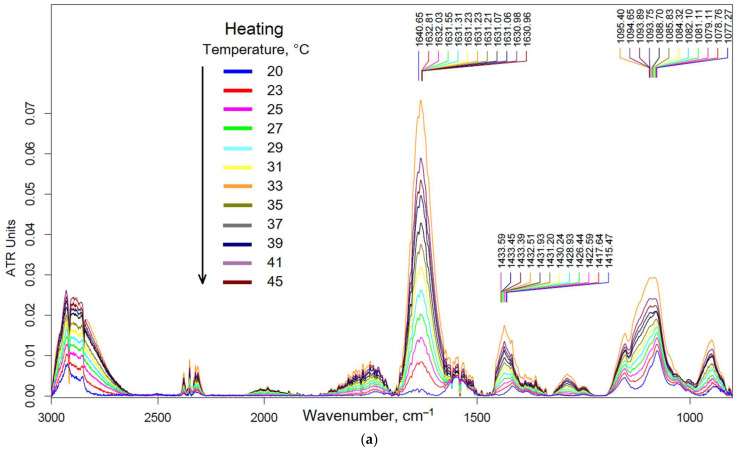

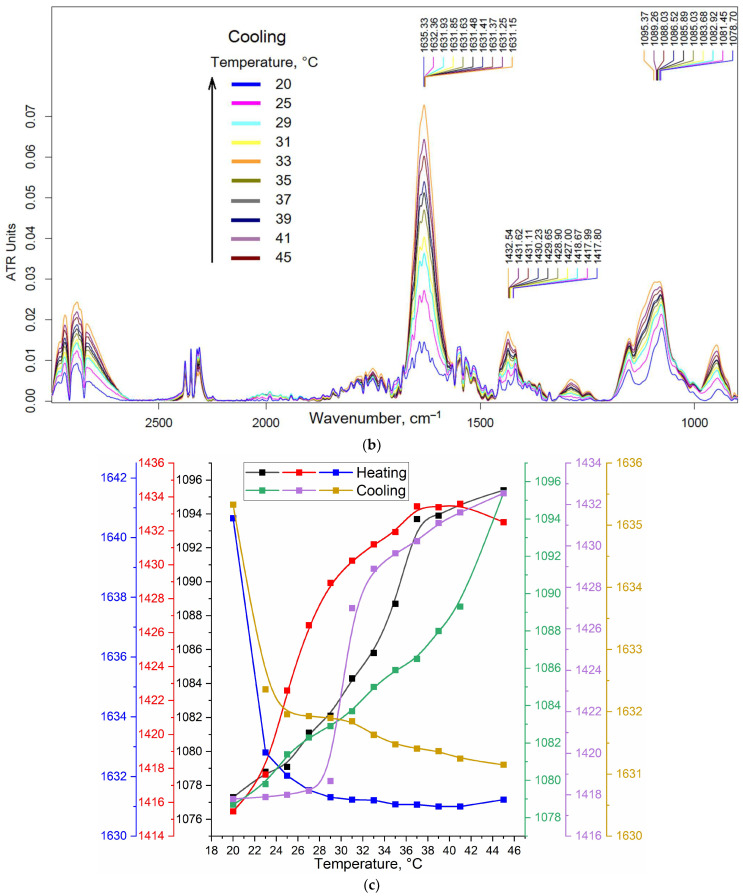
FTIR spectra of Chit5-PEG5 1% thermoreversible gel depending on temperature 20–45 °C: (**a**) heating process and (**b**) cooling process. (**c**) Corresponding dependencies of peak’s center mass on temperature.

Figure 3 shows the FTIR spectra of thermoreversible gel based on Chit5-PEG5 depending on temperature. The peak of 1630–1640 cm^−1^ characterizes the microenvironment of C=O bonds; the degree of carbonyl hydration decreases sharply at temperatures above 20 °C (blue and golden curves in Figure 3c) in the forward- and reverse-phase transition processes, since a shift in the peak to the low-frequency region is observed. The formation of the gel is reversible and is accompanied primarily by the removal of water molecules and polymer tangle and the formation of hydrophobic interactions. The peak at 1415–1434 cm^−1^ characterizes the C–H bending of chitosan and grafted PEG chains: the shift to the high-frequency region in the forward process occurs in a wide temperature range at 24–30 °C (red curve, Figure 3c) and in the reverse process at 29–30 °C (lilac curve, Figure 3c). This means that the folding of polymer chains is slow and reversible, and the unfolding of the tangle is fast and irreversible. The peak at 1095–1077 cm^−1^ corresponds to C–O–C fluctuations in the glucosamine units of chitosan, characterizes the final formation of a hydrophobic tangle at 34–38 °C (black curve, Figure 3c), when not only PEG chains but also chitosan chains stick together. Different temperatures of phase transitions for different functional groups are caused by the gelation step mechanism: first, a decrease in the degree of hydration of carbonyl groups; then, hydrophilic interactions of PEG chains and hydrophobic interactions between chitosan skeletons—physically, gelation corresponds to the phase transition according to changes in peak 1095–1077 cm^−1^ (C–O–C oscillations in the glucosamine units of chitosan) at 34–38 °C. Thus, the peak at 1100 cm^−1^ is the most analytically significant in the aspect of gelation, and the other 2 peaks are the pre-phase transition that the gel is about to form.

FTIR spectroscopy has previously been used to study the phase transitions of thermoreversible gels. For instance, in [29], titan–butoxide-based gel phase transition is presented: when heated at temperatures above 45 °C, bridges are formed due to opening oxygen atoms followed by hydrogen bond formation; when cooled, sol is regenerated. In relation to our work, this mechanism is not excluded. Water can play an important role in the formation of gel, as shown by the example of hydration of carbonyl groups. In another work [30], FTIR spectroscopy was used to determine functional groups (C–O–C bonds, as in our work for PEG chains) determining the thermoreversible properties of Pluronic-based gel: the C–O–C stretching bands tend to merge into a single broadband, shifting from 1106 to 1084 cm^−1^. Hydrophobic and hydrophilic interactions lead to shifts in the characteristic absorption bands in the FTIR spectra, for example, in the formation of micelles based on chitosan fatty acid [31,32,33]. Thus, FTIR spectroscopy provides molecular details of the gel formation.

#### 3.3.2. Fluorescence Spectroscopy of R6G in Reversible Gel

The use of a fluorescent label to track gelation provides valuable information about the mobility of polymer chains or the formation of hydrophobic sites, for example, using a covalent pyrene label [30]. In this paper, we propose a non-covalent label rhodamine 6G (R6G), which provides high sensitivity to the microenvironment changes and, thus, to the gelation process. An important aspect of the work is the study of thermoreversible gels, so it is necessary to find out whether the polymer really forms a gel at 37 °C.

Thermoreversible gels are formed by copolymers of hydrophilic and hydrophobic segments that can self-organize into polymer micelles in water. The mechanism of formation of thermogels consists of strengthening the hydrophobic interactions of chitosan chains at high temperature, but at the same time, finding the material in the form of a hydrogel but not aggregated precipitate (piece of polymer), due to the hydrophilic chains of PEG [34]. The fluorophore molecule dramatically changes the properties of fluorescence when the microenvironment changes, including when the fluorophore is embedded in the hydrophobic core. In [26], it was shown that the peak of the fluorescence emission of rhodamine 6G when loaded into chitosan nanocomposite widens and shifts to the long-wavelength region. The changes in the fluorescence parameters when fluorophores molecules are loaded into chitosan-based micelles were previously demonstrated by us [35,36].

**Figure 4:**
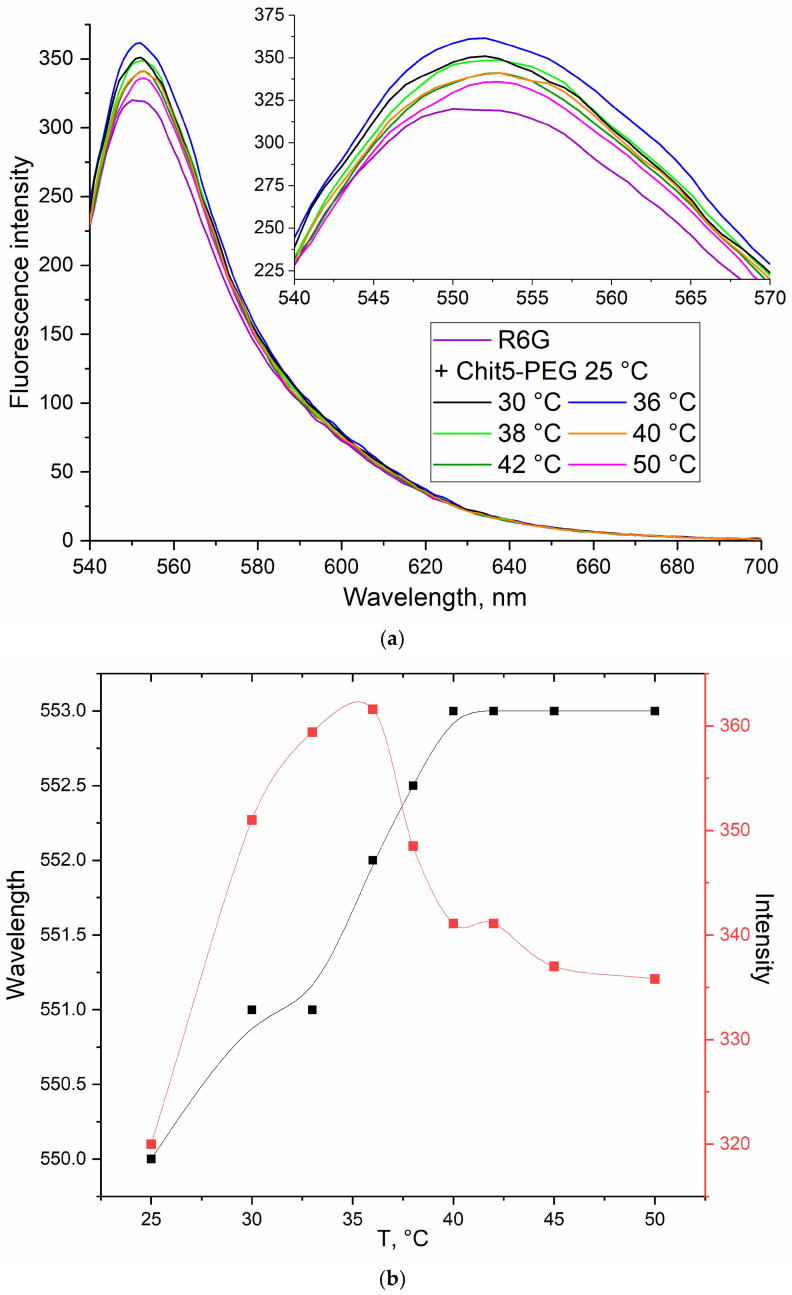
(**a**) Fluorescence emission spectra of R6G (0.1 μg/mL) + 1 mg/mL of polymer Chit5-PEG5 depending on temperature. (**b**) Corresponding dependencies of peak’s intensities and positions on the temperature. λ_exci_ = 520 nm. PBS (0.01 M, pH 7.4).

R6G was chosen as a model dye, which radically changes its fluorescence during the transition from an aqueous medium to a hydrophobic one, both the intensity of fluorescence emission and the λ_max_ [37,38]. Therefore, we study the thermograms of gels and monitor the phase transition via the fluorescence of rhodamine 6G, the fluorescence intensity of which increases when interacting with the Chit5-PEG5 polymer at 25–35 °C, and then, with further heating, the fluorescence intensity drops sharply due to the formation of a gel from disordered polymer chains around fluorophore molecules (Figure 4). The phase transition occurs actively at 35–40 °C and is accompanied by a shift in the maximum fluorescence emission into the long-wavelength region, which confirms an increase in the hydrophobicity of the R6G microenvironment. These data coincide with the phase transition curves obtained via FTIR spectroscopy for the peak at 1100 cm^−1^.

Thus, through a combination of methods, we demonstrated the thermal sensitivity of polymers and the potential ability to form gels for the prolonged release of drugs, including proteins and DNA. All chitosan-based polymers are prone to gelation, and chitosan modified with PEG is the best gel-forming polymer due to additional hydrophobic interactions of chitosan, since PEG chains are deposited outside the gel globula. All the presented polymers can be used as thermoreversible gels; however, pegylated chitosan is optimal.

### 3.4. DNA Interactions with Polymers

#### 3.4.1. FTIR Spectroscopy

**Figure 5:**
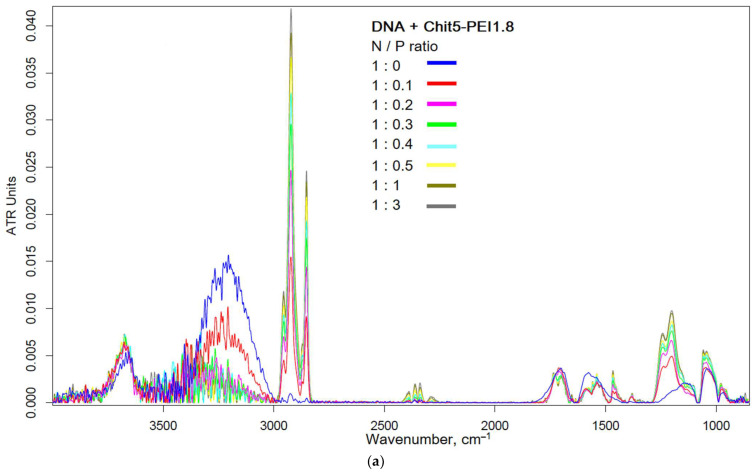

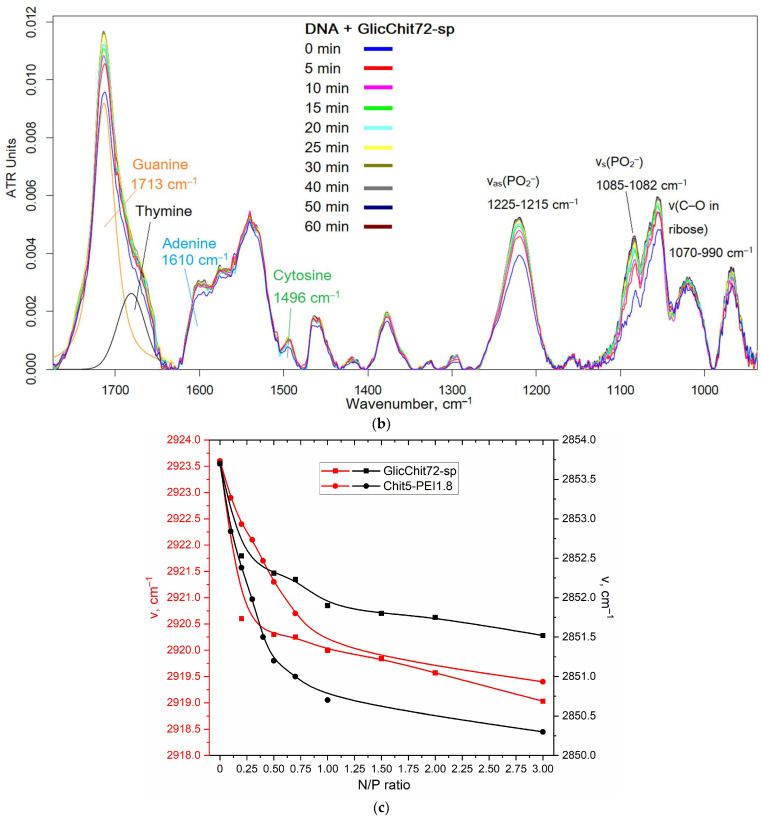
(**a**) FTIR spectra of plasmid DNA + Chit5-PEI1.8 polymer in different molar ratios. (**b**) FTIR spectra of plasmid DNA + GlicChit72-sp polymer during online incubation. (**c**) Dependences of FTIR peak’s positions on the (N) polymer/(P) DNA ratios. Graph (**b**) shows the decomposition of the curve into 2 components corresponding to guanine (orange) and thymine (black). PBS (0.01 M, pH 7.4). T = 37 °C.

FTIR spectroscopy characterizes not only DNA and cationic polymers separately but also provides valuable information about polyplexes (DNA–cationic polymer). The interaction of negatively charged phosphate groups with positively charged amino groups, as well as conformational changes, is reflected in the FTIR spectra [39,40]. Cationic polymers are designed to form polyplexes with DNA, which potentially leads to high transfection efficiency. The interaction of negatively charged DNA with positively charged polymers is reflected in the FTIR spectra, characterizing the microenvironment and the state of functional groups. Figure 5 shows the FTIR spectra of plasmid DNA in different molar ratios with the Chit5-PEI1.8 polycation and during online incubation with GlicChit72-sp. Characteristic peaks can be distinguished in the FTIR spectrum of DNA: guanine (1713 cm^−1^), thymine (1680 cm^−1^), adenine (1610 cm^−1^), cytosine (1496 cm^−1^), asymmetric and symmetric oscillations of phosphate groups 1220 and 1084 cm^−1^, C–O oscillations in ribose residues (1070–990 cm^−1^), O–H stretching (3400–3000 cm^−1^) in ribose, N–H stretching (3750–3600 cm^−1^) in nucleobases, as well as asymmetric and symmetric C–H oscillations (2923 and 2853 cm^−1^). The last peaks shift to the low-frequency region during the formation of DNA polyplexes with polycations (Figure 5c). The compaction of DNA into polyplexes is accompanied by an increase in the intensity of characteristic peaks, especially guanine, thymine and phosphate groups. When DNA binds to polycations, the intensity of the peak oscillations of phosphate groups increases (1220 cm^−1^, Figure 5). Figure 5b shows DNA binding over time: approximately 50% of polyplexes are formed in 5 min and almost completely for DNA in the form of polyplexes in 20–30 min. With a ratio of N/P = 1, most of the DNA is bound to a polycation, which indicates the possibility of using polymers as gene delivery tools.

Chit5-PEI1.8 was found to be the strongest polycation in the aspects of DNA binding, which was also discussed in the Introduction, minimizing the side effects of PEI but, at the same time, with high charge density. Chitosans modified with arginine or spermine show high DNA binding ability due to exposed amino groups, but the charge density is lower than that of Chit5-PEI1.8. PEGylated chitosan, due to the shielding of charges on chitosan by PEG chains, is less effective, but it should not be discounted since it is capable of forming thermoreversible gels.

#### 3.4.2. UV-Vis and Fluorescence Spectroscopy

**Figure 6:**
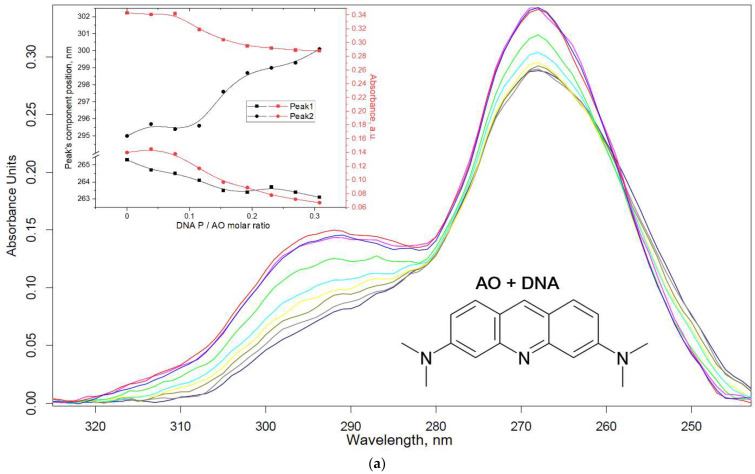

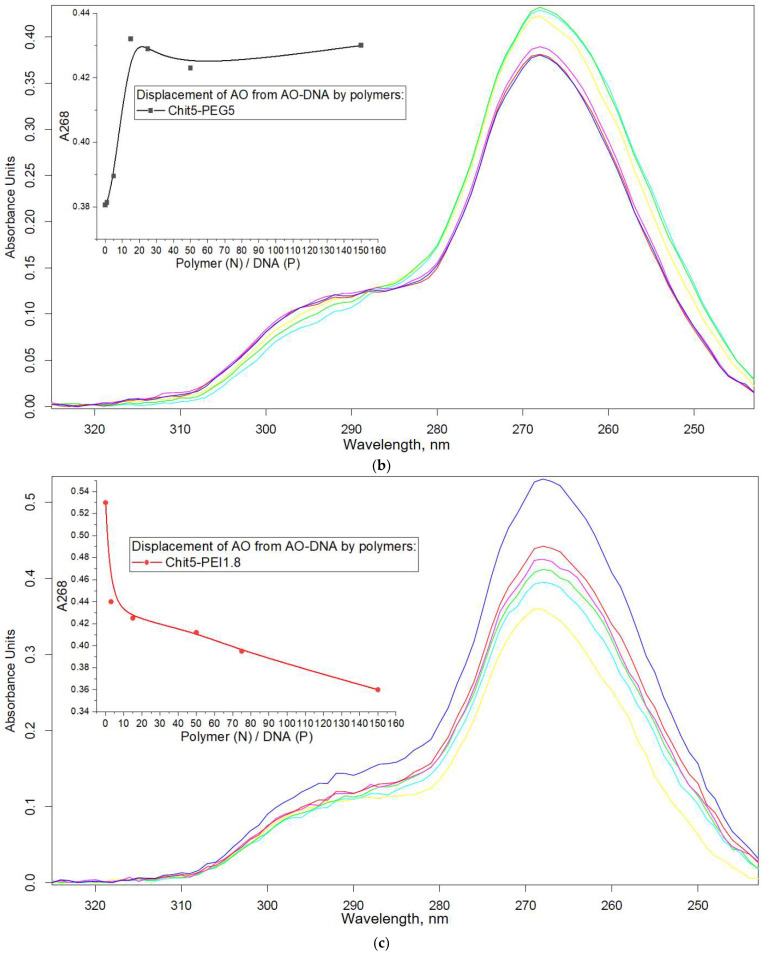

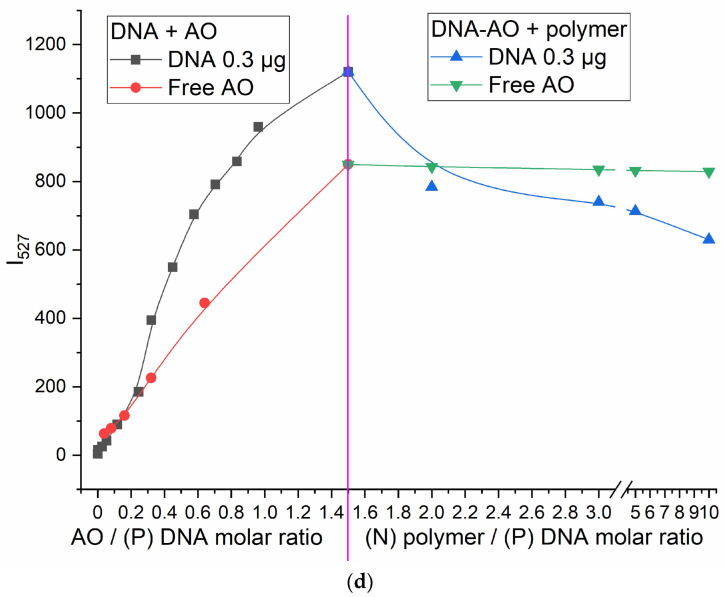
(**a**) UV spectra of AO in different ratios with DNA: from 0 (blue line) to 0.27–0.31 (gray and dark-blue lines) DNA molar excess. (**b**,**c**) UV spectra of AO-DNA (10:1 molar ratio) in different ratios with polymers. (**d**) The dependencies of maximum AO fluorescence during DNA titration followed by displacement of AO with polymer Chit5-Arg-GlucA. Synchronous fluorescence with δ = 30 nm, λ_max_ = 527 nm. PBS (0.01 M, pH 7.4). T = 22 °C.

Using FTIR spectroscopy, the formation of DNA polyplexes was confirmed. To determine the strength of the DNA–polymer complexes, the authors used UV and fluorescent spectroscopy with a model dye acridine orange (AO), which intercalates DNA (major intercalation of AO into polynucleotides G–C and A–T base pairs with minor external binding, K_AO_ = 2.69 × 10^4^ M^−1^ [41,42]) while changing its absorption and fluorescent properties (Figure 6). The interaction of AO with DNA leads to a decrease in the integral fraction of the component at 295 nm (Figure 6a) and a decrease in absorption at 295 and 268 nm, and this is also reflected in the fluorescence ignition of AO-DNA compared to free AO (Figure 6d). At a ratio of (P) DNA/(AO molecules) > 0.2, most of the dye is in a bound form. The subsequent displacement of AO from the DNA complex leads to an increase in absorption at 268 nm due to the release of AO (Figure 6b,c) and quenching of AO fluorescence when the DNA-AO complex is destroyed (Figure 6d). From the presented curves, it can be assumed that the polymer almost completely displaces AO at a 0.5–1 molar ratio of (N) polymer: AO, which confirms the strength of DNA–polymer polyplexes.

#### 3.4.3. DNA Electrophoresis

**Figure 7:**
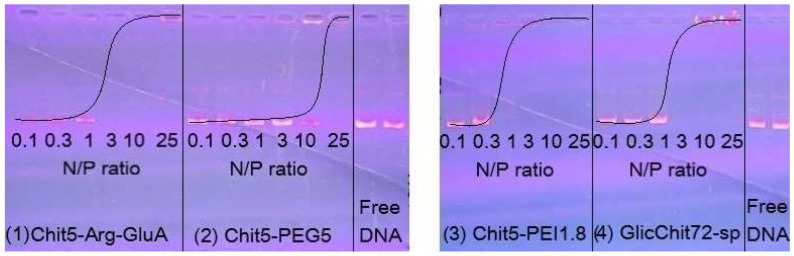
The results of electrophoresis for pDNA (6.8 kbase). V = 105 B. t = 100 min. DNA was labelled with ethidium bromide.

A qualitative confirmation of the formation of polyplexes from DNA (at which charge ratio all DNA will be in a complex with a polycation) was accomplished using electrophoregrams (Figure 7). Free DNA runs under the action of an electric field, and DNA in polyplexes remains at the start when the charge is neutralized by polycations. For polymers 1 and 4, the formation of polyplexes by more than 95% can be considered at a ratio of N/P ≅ 1. For pegylated chitosan 2, this value increases to 5 due to the shielding of charges by PEG chains, and in the case of polymer 3, it decreases to 0.5 due to the powerful PEI polymer (the gold standard of transfection). Thus, the copolymers developed are promising for the formation of polyplexes for gene prolonged delivery.

### 3.5. BSA and DNA Release from Gel

**Figure 8:**
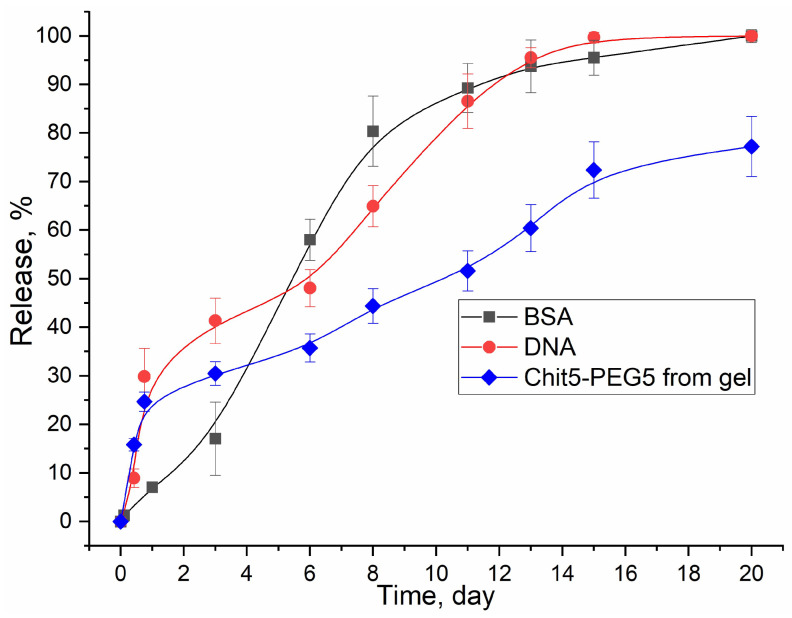
BSA (bovine serum albumin) and plasmid DNA release curves from 2% Chit5-PEG5. The blue curve indicates the destruction of the gel. 1MDa cut-off membrane. BSA was detected via A280. DNA was detected via A260. Chit5-PEG5 was detected using A235. C_BSA_ = 5 mg/mL. C_DNA_ = 0.2 mg/mL.

The cationic polymers described in this work at a concentration of 1–4% are capable of forming gels, and the thermoreversible property is most characteristic of pegylated chitosan 2. Negatively charged BSA (pI = 4.7) at pH = 7.4, used in the literature as a model object (peptide and protein properties), was selected for release from the gel, perfectly detected via A280. Figure 8 shows the curves of the release of BSA from the Chit5-PEG5 gel. BSA occupied the water channels of the hydrogel from which it would be released through diffusion along the concentration gradient between the gel matrix and release medium [2]. BSA is released by half in 5 days from the Chit5-PEG5 gel; full release is achieved in 18–20 days, which is comparable with the literature data [2]. At the same time, after 5 days, the gel is destroyed by up to 34%, and after 20 days, by 80% (release of chitosan particles). DNA from the gel is released in the form of DNA polymer polyplexes (according to the zeta potential of polyplexes vs. free DNA): the half-release period of polyplexes is approximately 7 days. Further, 80% of the DNA is released from the gel in about 10 days.

### 3.6. Fluorescence and Flow Cytomerty Approaches to Study DNA Polyplexes

**Figure 9:**
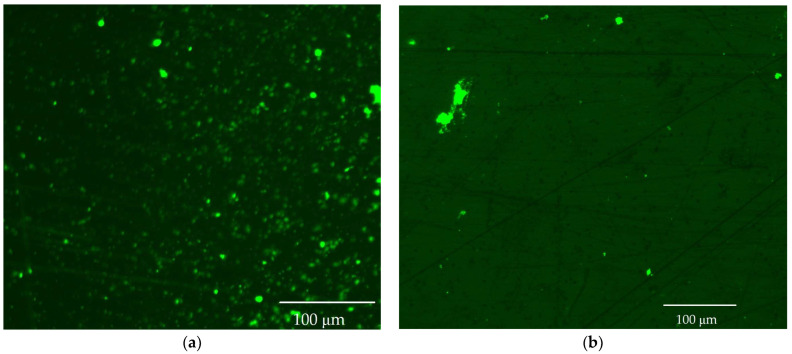
Fluorescence images of polyplexes: (**a**) DNA+ Chit5-Arg-GlucA, (**b**) DNA + Chit5-PEG5. DNA was labelled with AO.

Fluorescence imaging and flow cytometry can be used to study polyplexes. This approach is innovative but, at the same time, promising, since it has experimental prerequisites for determining the size of DNA-containing particles [43]. Figure 9 shows fluorescent images of DNA polyplexes and their aggregates. The particles of polyplexes formed and contained a fluorescent label specific to DNA. Flow cytometry allows you to visualize cells and particles with a size of at least 100–300 nm. The very fact of detecting such particles (Table 2) speaks in favor of the formation of large polyplexes. Table 2 shows diagrams of the distribution of DNA and DNA particles in the polyplex with the intensity of light scattering and fluorescence AO. Compared with free DNA, DNA particles in polyplex fall into the polyplex gate (high light-scattering intensity) by 10–30% more. For AO fluorescence in free DNA, there are two fractions: low fluorescence intensity (38%) and high fluorescence. At the same time, the DNA in the polyplex has only a high intensity of fluorescence. The polyplex gate (Table 2) obtains 59 and 39% of particles in the case of polymer Chit5-Arg-GlucA and Chit5-PEG5, respectively, which means polymer 1 binds more efficiently to DNA (more open charges), which is consistent with spectral data and electrophoresis data (Figure 7). These data confirm the formation of polyplex particles, which can potentially be used for developing drugs intended for gene delivery.

### 3.7. Studying the Properties of Gel Formation in Muscle Tissue in Chicken Model In Vitro

**Figure 10:**
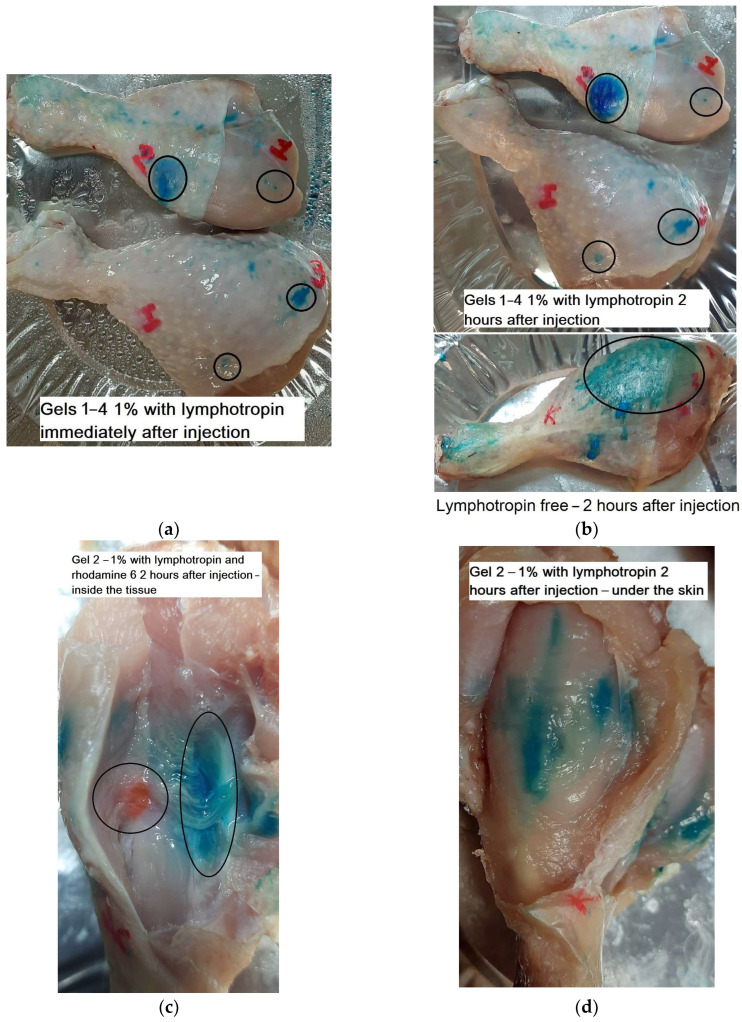

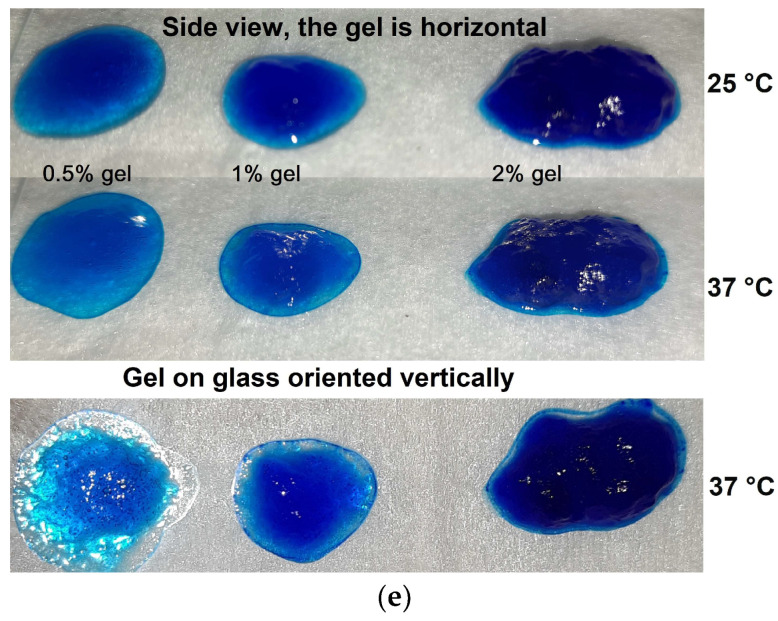
(**a**–**d**) Photos of chicken legs with injected dyes: blue—lymphotropin, orange-pink—rhodamine 6G; 1% thermogels. The samples were stored in an environment of sodium azide and ethanol to prevent spoilage. (**e**) Photos of thermogels with lymphotropin. T = 37 °C. Observation time: immediately after the introduction of dyes in the gel, after 2 h of incubation.

Chitosan-based gels containing medicinal preparations have already been used for in vivo trials [43], but the physicochemical aspects of the behavior of thermoreversible gel in tissue are the subject of the deep research in the development of new systems. The idea of the experiment is to study the formation of thermogel in chicken muscle tissue upon injection at 37 °C. Chicken legs, used as a model for studying the properties of gels and blurring of dyes, were proposed for the first time. There is very little information in the literature about these kinds of experiments. There is information about in vivo drug trials, but there are no data on the physico-chemical parameters of the decomposition of gels. Chicken muscle was chosen by us as a model of tissue, where a thermogel with the drug, including protein or DNA, will potentially be introduced. Lymphotropin was chosen due to its characteristic feature of blurring of dyes through tissues, which is significantly slowed down by thermogel, as well as electrostatic interaction with chitosan by sulfogroups. For comparison and control, rhodamine 6G is presented, because most of the experiments were carried out with it, but it is weakly blurring and poorly contrasted. Four gel samples of four polymers described in Table 1 were used. Rhodamine 6G and lymphotropin dye were used to visualize the gel. The status of the gel in chicken muscle tissue was studied after administration of the drug, 2 h and 2 days after administration. Figure 10 shows photos of the muscle tissue of chicken legs with injected dyes: blue—lymphotropin, orange-pink—rhodamine 6G. It can be seen that a gel is formed in the form of round spots at a certain density (different from the main tissue), which gradually releases the dye. After 2 days, partial preservation of the gel and blurring of about one-third of the tissue under study were observed, while the free dye not included in the gel was blurred in a few hours.

Thus, the formation of gel at 37 °C from sol at 25 °C is shown (Figure 10e), the most characteristic for a concentration of 1%. At concentrations below—the gel is formed weakly at high concentrations—the polymer gelation happens at room temperature. Thus, 1% gel injected into chicken muscle tissue significantly slows down the diffusion of the blue dye lymphotropin due to the formation of a solid clot. The obtained data are of interest in aspects of the creation of drugs in hydrogels with the function of ultra-long release.

## 4. Conclusions

Thermoreversible gels based on modified chitosans are of great interest for medical use in the field of drug and gene delivery. These liquid gels are injected intramuscularly at room temperature and harden quickly, which causes prolonged drug release, for example, DNA up to 30 days. For effective gene therapy, it is necessary to create DNA polyplexes with polycations, such as chitosan, polyethyleneglycol or copolymers, having the properties of biocompatibility, biodegradability and thermoreversibility. The PEI polycation is the gold standard of transfection; however, it is cytotoxic, so we proposed a combined variant of Chit5-PEI1.8 that is relatively safe and effectively binds DNA into a polyplex at the ratio of amino groups to phosphate (N/P > 0.3). Using the dyes acridine orange (AO) and rhodamine 6G (R6G), the physicochemical parameters of the formation of polyps were studied through molecular absorption and fluorescence spectroscopy. Phase transitions of thermogels and functional groups involved in solidification were studied using FTIR spectroscopy. Electrophoresis of DNA vs. DNA–polymers showed the formation of polyplexes at an N/P ratio from 0.3 to 3, depending on the type of polymer. For the first time, the formation of fluorescent polyplexes (400 nm) was confirmed using flow cytometry. The thermoreversible properties of chitosan-based copolymers are due to increased hydrophobic interactions of chitosan chains at elevated temperature. At the same time, in order for the gel to be a hydrogel, the copolymer must have hydrophilic fragments, for example, PEG, arginine and gluconic acid. Using dyes, the formation of gels is shown, the most striking at a 1% polymer concentration. The blurring of lymphotropin in chicken muscle tissue occurs by about 4 cm in diameter in an hour, while the lymphotropin in the gel is released slowly and weakly blurred to 1.5 cm. The release of DNA and the model BSA anion from the gel occurs by 50% in 5 days, by 80% in 8–10 days and by 95% and more in 13–15 days. The destruction of the gel by half occurs in 10 days, and complete destruction is achieved after 25–30 days (slower than the release of polyanions from the pores of the gel). This opens up prospects for the use of hydrogels with thermoreversible properties for the prolonged delivery of DNA and drugs.

## Figures and Tables

**Table 1:** Cationic polymers’ physico-chemical characteristics.

Code	Conjugate *	Modification Degree, % from the Number of Available Chitosan Amino Groups	Molecular Weight, kDa
1	Chit5-Arg-GlucA	Arg: 18 ± 3, GlucA: 21 ± 2	7 ± 1
2	Chit5-PEG5	7 ± 1 (Chit: PEG = 1:2 molar ratio)	15 ± 3
3	Chit5-PEI1.8	4 ± 1 (Chit: PEI = 3:1 molar ratio)	17 ± 5
4	GlicChit72-sp	16 ± 4 (Chit: sp ≅ 1:35 molar ratio)	85 ± 14

* 5, 1.8 and 72 (±5)—is molecular weight (kDa) of initial polymers.

**Table 2:** Flow cytometry diagrams for DNA polyplexes. DNA was labelled with AO. AO fluorescence is shown in FITC-A channel. Polyplex and positive—the area of large particles.

DNA Form	Side Scattering (SSC-A) vs. Front Scattering (FSC-A)	Side Scattering (SSC-A) vs. AO Channel (FITC-A)	Distribution of Particles by AO-Fluorescence (Blue–High Intensity in Polyplexes, Red–Low in Debris
DNA + Chit5-Arg-GlucA	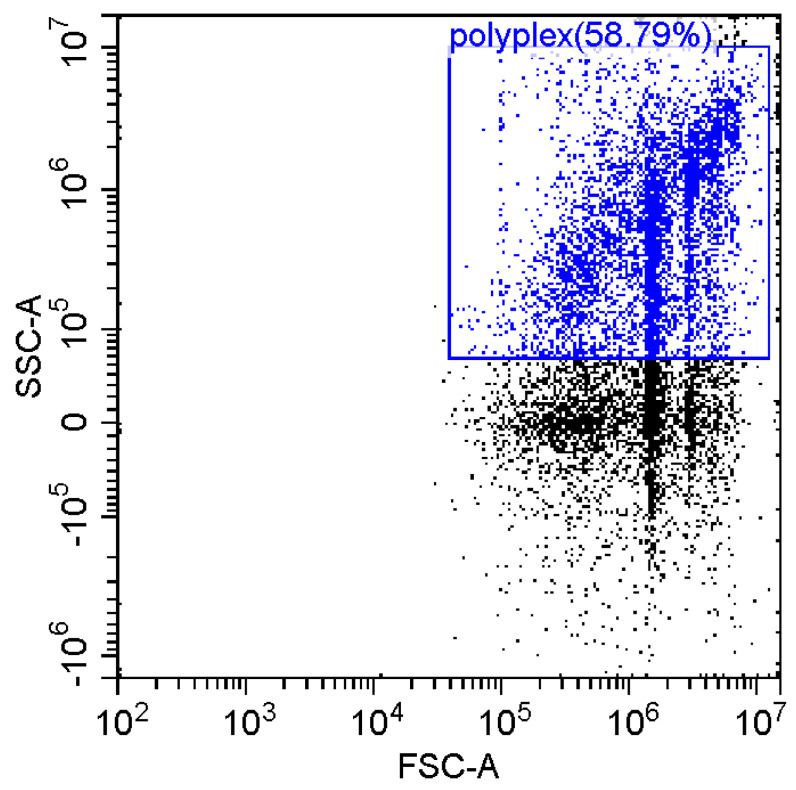	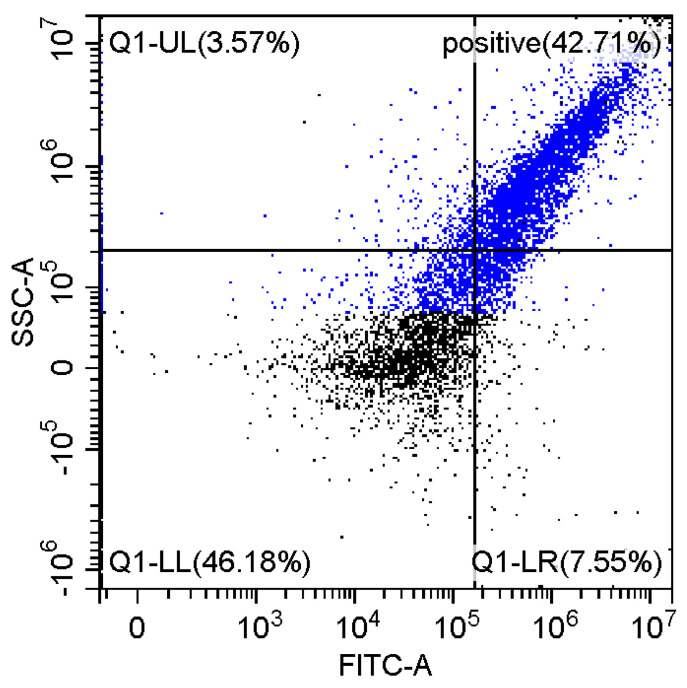	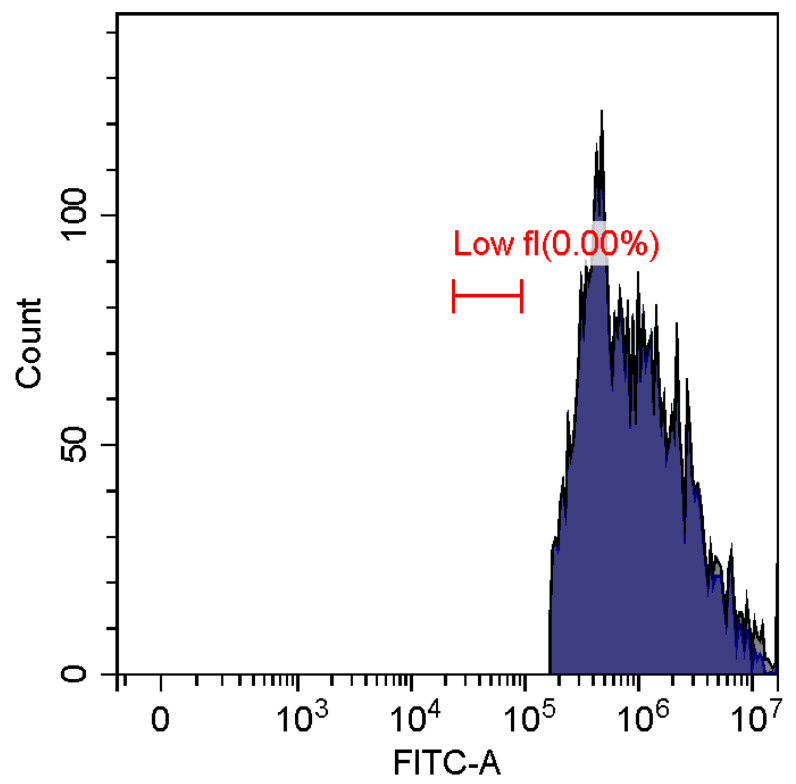
DNA + Chit5-PEG5	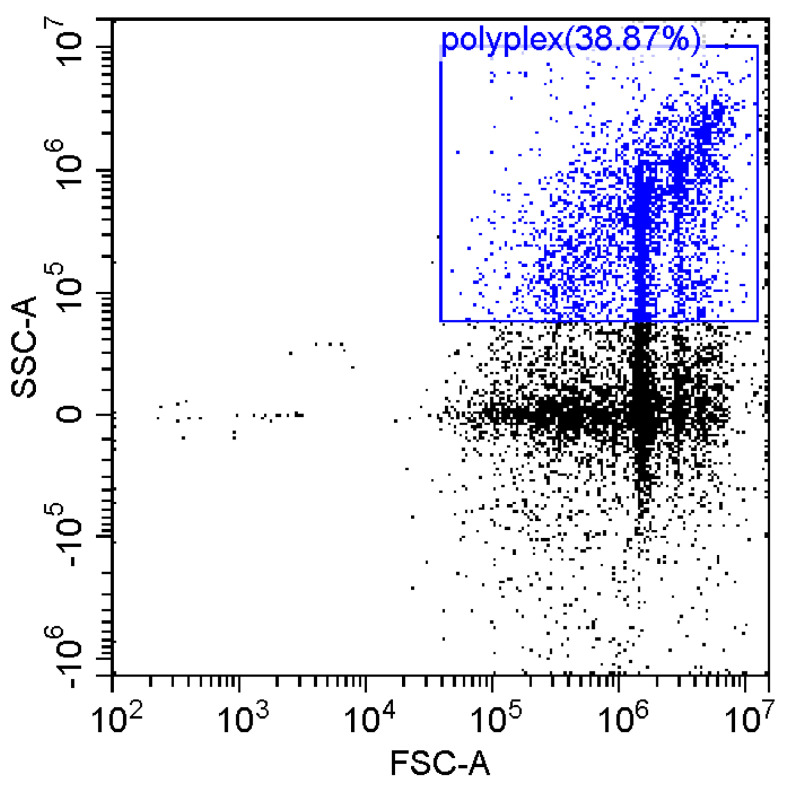	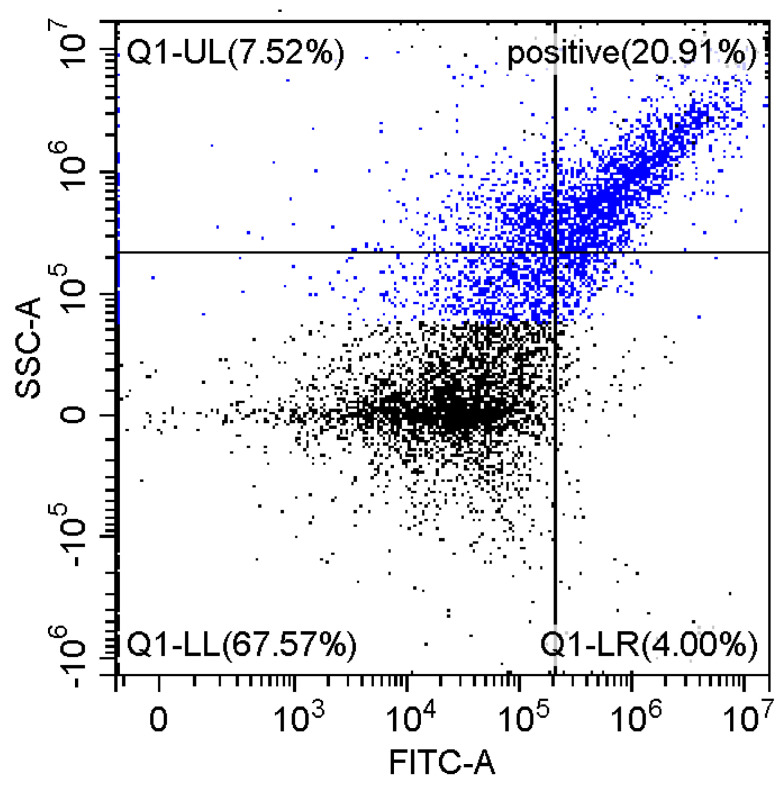	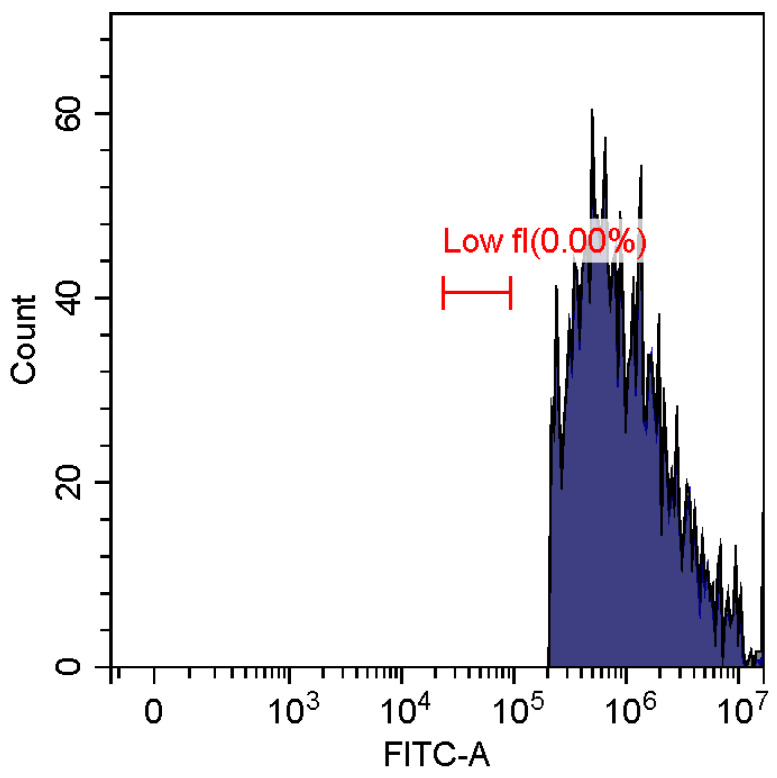
DNA free	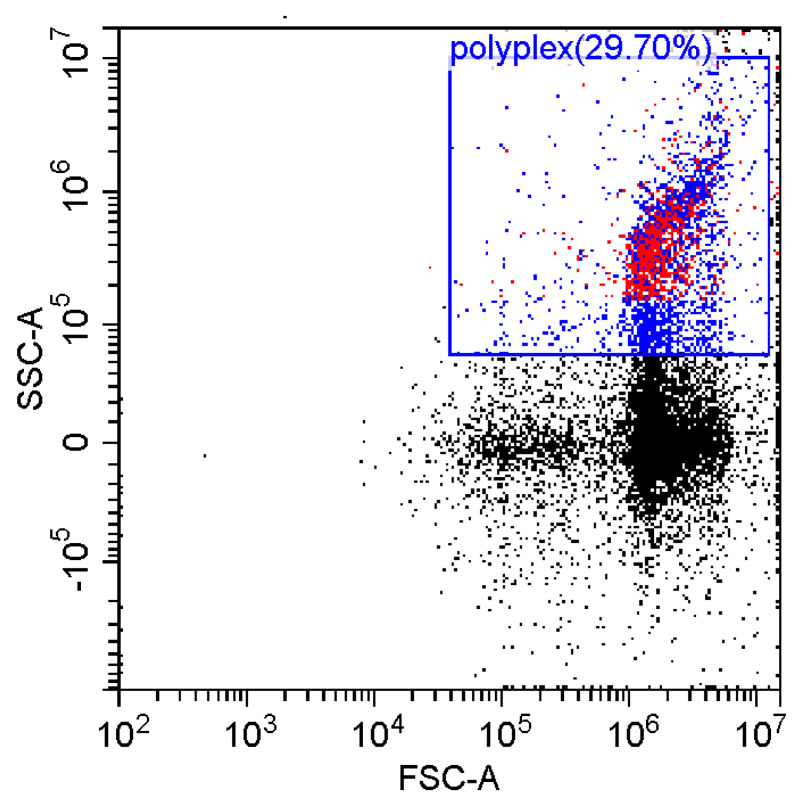	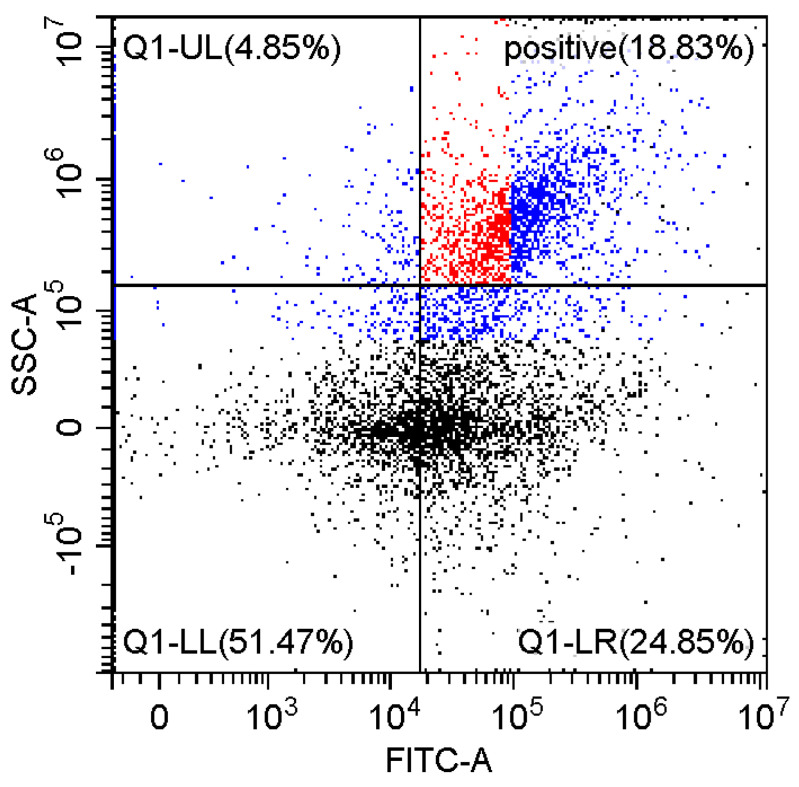	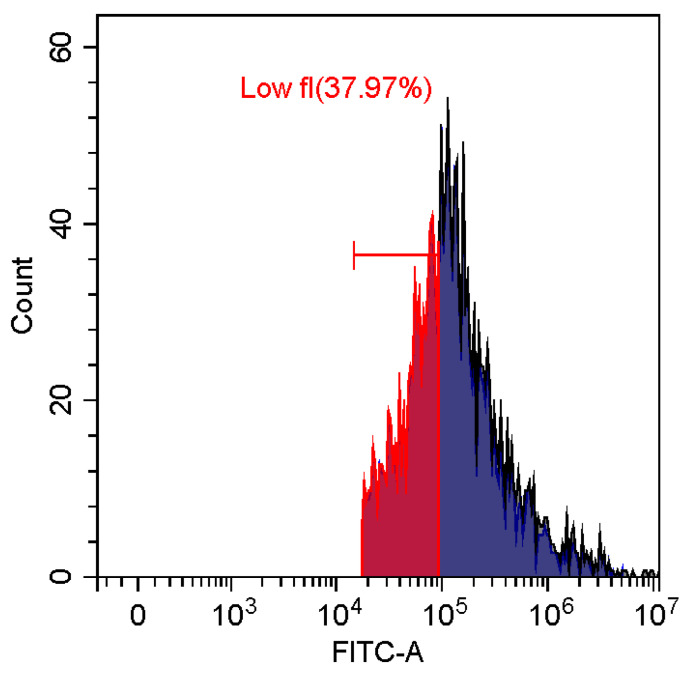

## Data Availability

The data presented in this study are available in the main text.

## Abbreviations

AO	acridine orange
Arg	arginine
CDI	carbonyldiimidazole
Chit	chitosan
EDC	1-Ethyl-3-(3-dimethylaminopropyl) carbodiimide
GlicChit	glycol chitosan
GlucA	gluconic acid
MM	molar mass
N	aminogroup in polymers
NHS	N-hydroxysuccinimide
P	phoshate group in DNA
PEG	polyethyleneglycol
PEI	polyethyleneimine
